# Supplementary figures and images for: Species and size diversity in protective services offered by coral guard-crabs
Source: PeerJ. 2014 Sep 30;2:e574. doi: 10.7717/peerj.574 (PMC4183949; doi:10.7717/peerj.574)

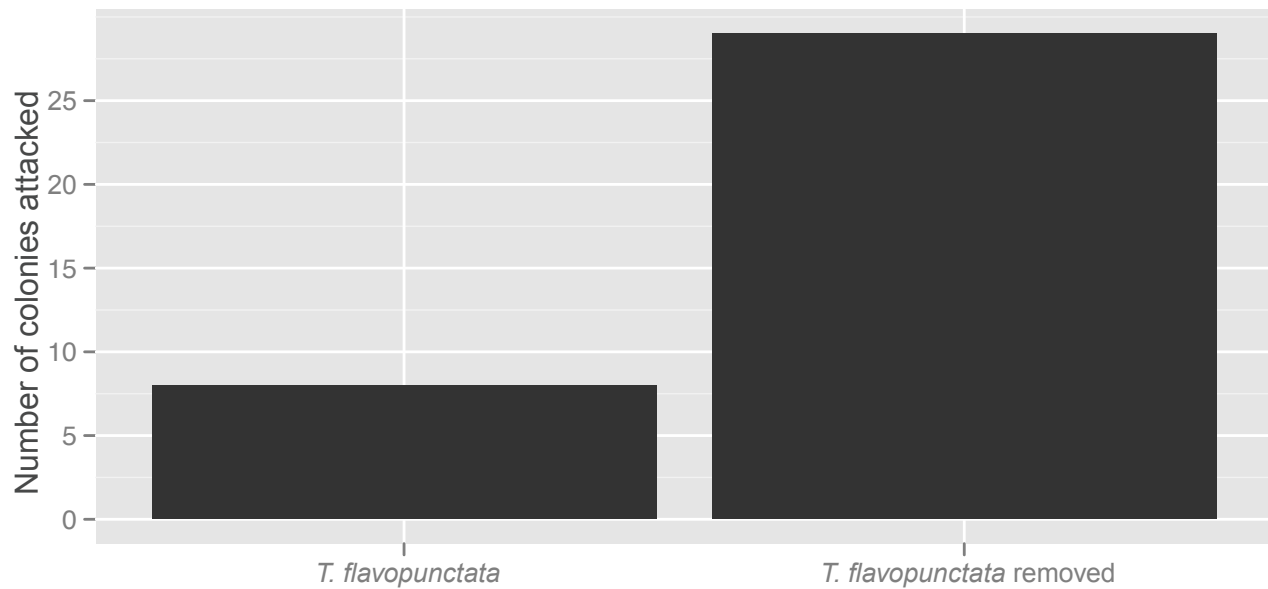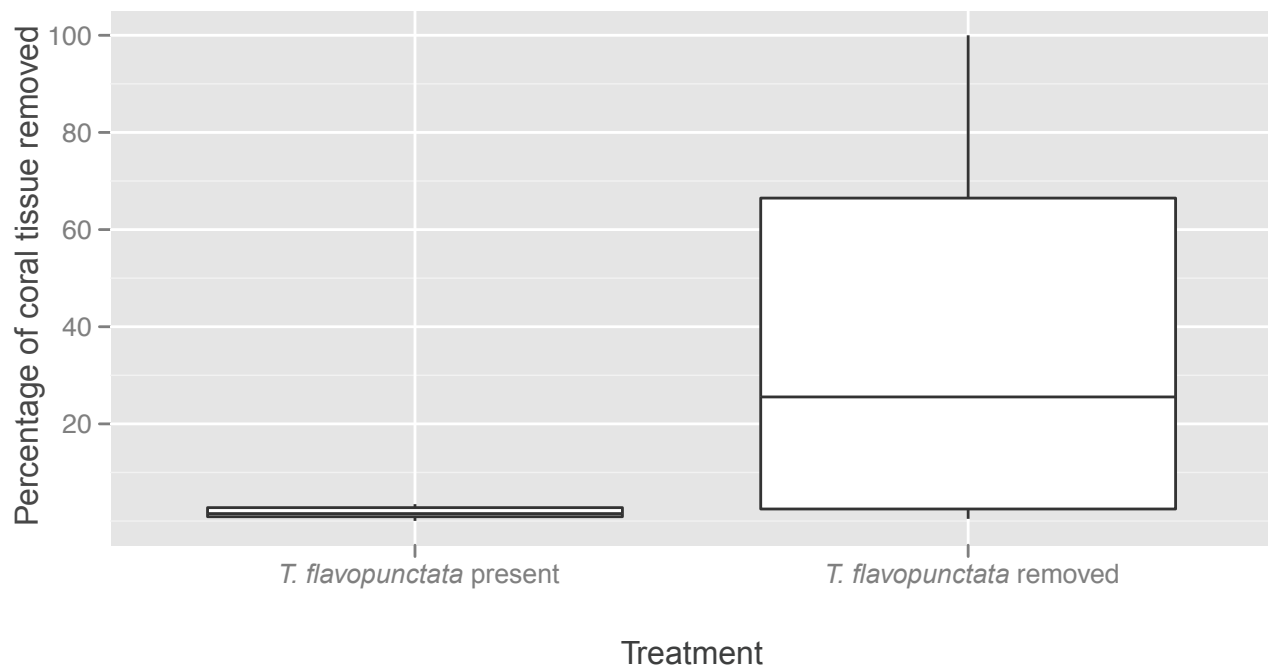

Supplement: Supplemental Information 1 [file peerj-02-574-s001.pdf]
